# Angular measures and Birkhoff orthogonality in Minkowski planes

**DOI:** 10.1007/s00010-020-00715-4

**Published:** 2020-02-22

**Authors:** Márton Naszódi, Vilmos Prokaj, Konrad Swanepoel

**Affiliations:** 1 Department of Geometry, Alfréd Rényi Institute of Mathematics, Eötvös Loránd University, Budapest, Hungary; 2 Department of Probability Theory and Statistics, Eötvös Loránd University, Budapest, Hungary; 3 Department of Mathematics, London School of Economics, London, UK

**Keywords:** Primary 52A21, Secondary 28A75, 46B20, Angle measure, Birkhoff orthogonality, Minkowski space, Normed space, Radon planes

## Abstract

Let *x* and *y* be two unit vectors in a normed plane $\mathbb {R}^2$. We say that *x* is *Birkhoff orthogonal* to *y* if the line through *x* in the direction *y* supports the unit disc. A *B-measure* (Fankhänel in Beitr Algebra Geom 52(2):335–342, 2011) is an angular measure $\mu$ on the unit circle for which $\mu (C)=\pi /2$ whenever *C* is a shorter arc of the unit circle connecting two Birkhoff orthogonal points. We present a characterization of the normed planes that admit a B-measure.

## Introduction

Let *K* be an origin-symmetric *convex body* in the plane, that is, a compact convex set with non-empty interior in $\mathbb {R}^2$, and consider the normed plane $(\mathbb {R}^2,\left\Vert \cdot \right\Vert _{K})$, where $\left\Vert x\right\Vert _{K}=\min \left\{ \lambda >0\;:\;x\in \lambda K\right\}$ for any $x\in \mathbb {R}^2$. Then *K* is the *unit ball* of the norm, and its boundary ${{\,\mathrm{bd}\,}}K$ the *unit circle*.

Let $x,y\in {{\,\mathrm{bd}\,}}K$ be two unit vectors in $\mathbb {R}^2$. We say that *x* is *Birkhoff orthogonal* to *y*, and denote it by $x\dashv y$, if $\left\Vert x\right\Vert _{K}\le \left\Vert x+ty\right\Vert _{K}$ for all $t\in \mathbb {R}$. Geometrically, this means that the line through the point *x* in the direction *y* supports the unit ball *K*. In general, Birkhoff orthogonality is not a symmetric relation. Normed planes where Birkhoff orthogonality is symmetric are called *Radon planes* and the boundaries of their unit balls *Radon curves* (see the survey [5]).

A Borel measure $\mu$ on ${{\,\mathrm{bd}\,}}K$ is called an *angular measure*, if $\mu ({{\,\mathrm{bd}\,}}K)=2\pi$, $\mu (X)=\mu (-X)$ for every Borel subset *X* of ${{\,\mathrm{bd}\,}}K$, and $\mu$ is *continuous*, that is, $\mu (\{x\})=0$ for every $x\in {{\,\mathrm{bd}\,}}K$. There always exists an angular measure on ${{\,\mathrm{bd}\,}}K$, such as the one-dimensional Hausdorff measure on ${{\,\mathrm{bd}\,}}K$ normalized to $2\pi$, but an arbitrary angular measure does not necessarily have any relation to the geometry of $(\mathbb {R}^2,\left\Vert \cdot \right\Vert _{K})$. A natural problem then is to find angular measures with interesting geometric properties. For instance, Brass [2] showed that whenever the unit ball is not a parallelogram, there is an angular measure in which the angles of any equilateral triangle are equal. This type of angular measure is very useful in studying packings of unit balls [2, 8]. Angular measures with other properties have been proposed; see the survey [1, Section 4] for an overview. An angular measure $\mu$ is called a *B-measure* [3] if $\mu (C)=\pi /2$ for every closed arc *C* of ${{\,\mathrm{bd}\,}}K$ that contains no opposite points of ${{\,\mathrm{bd}\,}}K$, and whose endpoints *x* and *y* satisfy $x\dashv y$.

The main result of this note (Theorem 1) is a characterization of the normed planes $(\mathbb {R}^2,\left\Vert \cdot \right\Vert _{K})$ which admit a B-measure. In order to formulate this theorem, we need to introduce two subsets of ${{\,\mathrm{bd}\,}}K$.

We call a point *x* in ${{\,\mathrm{bd}\,}}K$ an *Auerbach point*, if there is a $y\in {{\,\mathrm{bd}\,}}K$ such that $x\dashv y$ and $y\dashv x$. In this case we say that *x* and *y* form an *Auerbach pair*. It is well known that Auerbach points exist for any norm [9, Section 3.2]. We denote the set of Auerbach points of *K* by *A*(*K*). Note that *A*(*K*) is a closed subset of ${{\,\mathrm{bd}\,}}K$. We denote the union of open non-degenerate line segments contained in ${{\,\mathrm{bd}\,}}K$ by *E*(*K*).

### Theorem 1

Let *K* be an origin-symmetric convex body in $\mathbb {R}^2$. Then there is a B-measure on ${{\,\mathrm{bd}\,}}K$ if, and only if, the set $A(K){\setminus } E(K)$ is uncountable.

This is a strengthening of a result of Fankhänel [3, Theorem 1], where the existence of a B-measure is shown under the condition that $A(K){\setminus } E(K)$ contains an arc. (Fankhänel does not explicitly exclude line segments, but it is clear that they have to be excluded, as line segments in *A*(*K*) necessarily have measure 0 for any B-measure; see Lemma 3.) We prove Theorem 1 in Section 2, where we also present a smooth, strictly convex, centrally symmetric planar body *K* such that *A*(*K*) is the union of two disjoint copies of the Cantor set and a countable set of isolated points (Example 4). Thus, *A*(*K*) is of Lebesgue measure zero and yet, by Theorem 1, there is a B-measure on ${{\,\mathrm{bd}\,}}K$.

We recall that a subset of a topological space is called *perfect* if it is closed and has no isolated point. Recall that the *support* ${{\,\mathrm{supp}\,}}(\mu )$ of a Borel measure $\mu$ on a topological space *X* is the set of all $x\in X$ such that all open sets containing *x* have positive $\mu$-measure. It is easy to see that the support of any continuous measure is a perfect set. In the proof of Theorem 1, we rely on the following converse for $X=[0,1]$.

### Proposition 2

Let $H\subset [0,1]$ be a non-empty, perfect set. Then there is a continuous probability measure on [0, 1] whose support is *H*.

This is a well-known result holding more generally for any separable complete metric space [6, Chapter II, Theorem 8.1], but for the convenience of the reader we present an explicit construction for this special case in Section 3. It is well known that every non-empty perfect set is uncountable [7, Theorem 2.43] and every uncountable Borel set contains a perfect set [4, Section 6B]. (There is an even larger class, the analytic sets, with this property [4], but we will only need it for $F_\sigma$ sets).

## The Auerbach set and B-measure

Given two non-opposite points $a,b\in {{\,\mathrm{bd}\,}}K$, we denote by $\sphericalangle (a,b)$ the closed arc from *a* to *b* that does not contain any opposite pairs of points. We denote the closed line segment with endpoints $a,b\in \mathbb {R}^2$ by [*a*, *b*].

### Lemma 3

Let *K* be an origin-symmetric convex body in $\mathbb {R}^2$ and $\mu$ be a B-measure on ${{\,\mathrm{bd}\,}}K$. Then ${{\,\mathrm{supp}\,}}(\mu )\subseteq A(K){\setminus } E(K)$.

### Proof

Let $x\in E(K)$. Then $x\in [x^-,x^+]\subset {{\,\mathrm{bd}\,}}K$ for some $x^-,x^+$ with $x,x^-,x^+$ distinct. Let $y\in {{\,\mathrm{bd}\,}}K$ be parallel to $[x^-,x^+]$. Since $x^-,x^+\dashv y$, we have $\mu ([x^+,y])=\mu ([x^-,y])=\pi /2$, hence $\mu ([x^-,x^+])=0$ and $x\notin {{\,\mathrm{supp}\,}}(\mu )$.

Next, let $x\in {{\,\mathrm{bd}\,}}K{\setminus } A(K)$. Let $y_1,y_2\in {{\,\mathrm{bd}\,}}K$ such that $x\dashv y_1$ and $y_2\dashv x$. Then $y_1\ne y_2$. By possibly replacing $y_2$ by $-y_2$, we assume without loss of generality that $y_1$ and $y_2$ are in the same open half plane bounded by the line *ox*. By possibly replacing *x* by $-x$, we may also assume without loss of generality that $y_2$ and *x* are in the same open half plane bounded by $oy_1$. Let $x_1$ and $x_2$ be points on the same side of $oy_1$ as *x* such that $y_1\dashv x_1$ and $x_2\dashv y_2$. Then $x_1,x_2\ne x$. Because $y_2$ is between *x* and $y_1$, we have that $x_1$ and $x_2$ are in opposite open half planes bounded by *ox*. As above, since $\mu$ is a B-measure, $\mu (\sphericalangle (x_1,x_2))=\mu (\sphericalangle (x,x_1))=\mu (\sphericalangle (x,x_2))=0$, hence $x\notin {{\,\mathrm{supp}\,}}(\mu )$. $\square$

### Proof of Theorem 1

Let $\mu$ be a B-measure on ${{\,\mathrm{bd}\,}}K$. Then ${{\,\mathrm{supp}\,}}(\mu )$ is a perfect set, hence uncountable, and Lemma 3 gives that $A(K){\setminus } E(K)$ is uncountable.

Conversely, assume that $\widetilde{A}:=A(K){\setminus } E(K)$ is uncountable. We next find an appropriate perfect subset of $\widetilde{A}$ and use Proposition 2 to define a B-measure on ${{\,\mathrm{bd}\,}}K$. We first need to define an auxiliary map $\phi :\widetilde{A}\rightarrow A(K)$ by setting $\phi (x)$ to be the first $y\in A(K)$ in the positive direction along ${{\,\mathrm{bd}\,}}K$ from *x* so that $x\dashv y$ and $y\dashv x$. Then $\phi$ is monotone, but not necessarily injective. However, if $\phi (x_1)=\phi (x_2)$, then $x_1\dashv y$ and $x_2\dashv y$, as well as $x_1$ and $x_2$ being on the same side of line *oy*. Thus $[x_1,x_2]$ is a line segment on ${{\,\mathrm{bd}\,}}K$. Since the set

$$\begin{aligned} E^{\prime }(K):=\left\{ y\in {{\,\mathrm{bd}\,}}K\;:\;K \text { has more than one supporting line at } y\right\} \end{aligned}$$

is countable, it follows that for any given $y\in A(K)$, there are at most two values of $x\in \widetilde{A}$ such that $\phi (x)=y$, and there are at most countably many $y\in A(K)$ for which there is more than one $x\in \widetilde{A}$ such that $\phi (x)=y$. In particular, $\phi$ is a Borel measurable map.

We next find an appropriate arc $\sphericalangle (a,b)$ such that $\sphericalangle (a,b)\cap \widetilde{A}$ is uncountable. For any $x\in {{\,\mathrm{bd}\,}}K$, let $x^+$ denote the first element of $\widetilde{A}$ in the positive direction from *x*, and let $x^-$ be the first element of $\widetilde{A}$ in the negative direction from *x*. (If $x\in \widetilde{A}$ then $x=x^-=x^+$).

Let $\overline{E}(K)$ denote the union of the *closed* line segments on ${{\,\mathrm{bd}\,}}K$. Then $\overline{E}(K)$ is the union of *E*(*K*) with a countable set. Observe that for any $p\in {{\,\mathrm{bd}\,}}K$, the set $\phi ^{-1}(p)$ contains at most two points. Thus, $\phi ^{-1}(E^{\prime }(K))$ is countable. Moreover, $\phi ^{-1}(\bar{E}(K))$ is countable, since $\phi$ takes at most one value on an open line segment on ${{\,\mathrm{bd}\,}}(K)$. Fix an element

$$\begin{aligned} \begin{aligned} a\in A(K)\setminus \left[ \overline{E}(K)\cup E^{\prime }(K)\cup \phi ^{-1}\left( \bar{E}(K)\cup E^{\prime }(K)\right) \right] , \end{aligned} \end{aligned}$$

and let $b=\phi (a)$. Since $a\notin E^{\prime }(K)$, the only two points of ${{\,\mathrm{bd}\,}}(K)$ that form an Auerbach pair with *a* are $\pm b$. Since $a\notin \overline{E}(K)$, the only two points of ${{\,\mathrm{bd}\,}}(K)$ that form an Auerbach pair with *b* are $\pm a$. Since $a\notin \phi ^{-1}(E(K))$, we have $b\in A(K){\setminus } E(K)$. It follows that $\phi (b)=-a, \phi (-a)=-b$ and $\phi (-b)=a$.

We also obtain that $\sphericalangle (a,b)\cap \widetilde{A}$ or $\sphericalangle (b,-a)\cap \widetilde{A}$ is uncountable. Thus we may assume without loss of generality that $\sphericalangle (a,b)\cap \widetilde{A}$ is uncountable, where $\phi (a)=b$ and $\phi (b)=-a$, so it contains a perfect set, and by Proposition 2 there is a continuous probability measure $\nu$ on the Borel sets of ${{\,\mathrm{bd}\,}}K$ with ${{\,\mathrm{supp}\,}}(\nu )\subseteq \sphericalangle (a,b)\cap \widetilde{A}$. We use $\nu$ to define the B-measure as follows. For any Borel set $S\subseteq {{\,\mathrm{bd}\,}}K$, let

1 $$\begin{aligned} \mu (S):=\frac{\pi }{2}\big [\nu (S)+\nu (-S)+\nu (\phi ^{-1}(S))+\nu (\phi ^{-1}(-S))\big ]. \end{aligned}$$

Then $\mu$ is clearly an angular measure. Showing that $\mu$ is a B-measure is somewhat technical, mainly because $\dashv$ is not in general a symmetric relation. Let $x, y\in {{\,\mathrm{bd}\,}}K$ with $x\dashv y$. We have to show that $\mu (\sphericalangle (x,y))=\pi /2$. After possibly replacing *x* by $-x$ and *y* by $-y$, we may assume that $x\in \sphericalangle (a,b)\cup \sphericalangle (b,-a)$ and $y\in \sphericalangle (a,b)\cup \sphericalangle (b,-a)$.

- **Case 1:** $x\in \sphericalangle (a,b)$. Then either $y\in \sphericalangle (a,b)$ or $y\in \sphericalangle (b,-a){\setminus }\{b\}$.
- **Case 1.1:** $y\in \sphericalangle (a,b)$. There are two cases depending on the relative position of *x* and *y*.
- **Case 1.1.1:** $x\in \sphericalangle (a,y)$. Since $a\notin \overline{E}(K)$, we obtain $x=a$, and since $a\notin E'(K)$, we obtain $y=b$. Hence, $\mu (\sphericalangle (x,y))=\pi /2$ as required.
- **Case 1.1.2:** $x\in \sphericalangle (y,b)$. Since $b\notin E'(K)$, we obtain $y=a$, and since $b\notin \overline{E}(K)$, we obtain $x=b$, and again $\mu (\sphericalangle (x,y))=\pi /2$.
- **Case 1.2:** $y\in \sphericalangle (b,-a){\setminus }\{b\}$. In order to show that $\mu (\sphericalangle (x,y))=\pi /2$, it will be sufficient to show that $\phi ^{-1}(\sphericalangle (b,y))$ equals $\sphericalangle (a,x)\cap \widetilde{A}$ up to $\nu$-measure 0. In fact, we show that 2

First, let $p\in \phi ^{-1}(\sphericalangle (b,y^+)){\setminus } \phi ^{-1}(E(K))$. Then $\phi (p)\in \sphericalangle (b,y^+)$ and $p\in \widetilde{A}$. Without loss of generality, $\phi (p)\ne b,y^+$, and we want to show that $p\in \sphericalangle (a,x)$. Clearly, $p\in \sphericalangle (a,b)$. Suppose that $p\in \sphericalangle (x,b)$ and $p\ne x$. It follows from $p\dashv \phi (p)$ and $x\dashv y$ that $\phi (p)\notin \sphericalangle (b,y){\setminus }\{y\}$, since otherwise $p=x$. Therefore, $\phi (p)\in \sphericalangle (y,y^+)$. However, since $\phi (p),y^+\in \widetilde{A}$, we obtain the contradiction $\phi (p)=y^+$. Therefore, $p\notin \sphericalangle (x,b){\setminus }\{x\}$, and it follows that $p\in \sphericalangle (a,x)$, which finishes the proof of the $\subseteq$-inclusion of (2).

For the opposite inclusion, we assume without loss of generality that $p\in \sphericalangle (a,x)\cap \widetilde{A}$ and $\phi (p)\ne b,y^+$. Suppose that $\phi (p)\notin \sphericalangle (b,y^+)$. Then $y^+\in \sphericalangle (b,\phi (p)){\setminus }\{\phi (p)\}$. By considering $p\dashv \phi (p)$ and $x\dashv y$, we obtain that $p=x$, so $p\in \{x\}\cap \widetilde{A}$. This proves the $\supseteq$-inclusion of (2).

- **Case 2:** $x\in \sphericalangle (b,-a)$. This case is very similar to Case  1 and we only summarize the argument.
- **Case 2.1:** $y\in \sphericalangle (b,-a)$. As in Case 1.1, we use $a,b\notin E'(K)\cup \overline{E}(K)$ to obtain that $\{x,y\}=\{a,b\}$.
- **Case 2.2:** $y\in \sphericalangle (a,b)$. In an almost identical way as in Case 1.2, we can show that

from which it follows that $\nu (\sphericalangle (b,x))=\nu (\sphericalangle (a,y))$, hence $\mu (\sphericalangle (x,y)=\pi /2$ by (1).

This completes the proof of Theorem 1. $\square$

### Example 4

We present a smooth, strictly convex, origin-symmetric planar body *K* such that *A*(*K*) is the union of two disjoint copies of the Cantor set and a countable set of isolated points.

First, let *D* denote the Euclidean unit disk centered at the origin, and let *C* be the shorter arc connecting the two points whose angles with the positive *x* axis are $-\pi /4$ and $\pi /4$. Let $C_0$ denote the Cantor set in *C*. Now, $C_0$ can be written as

$$\begin{aligned} C_0=C{\setminus }\bigcup _{n=1}^{\infty }I_n, \end{aligned}$$

where the $I_n$ are disjoint open arcs in *C*.

For each $n\in \mathbb {Z}^+$, we construct a smooth and strictly convex curve $C_n$ connecting the two endpoints of $I_n$ with the following properties.

1. $C_n$ has the same tangents at the endpoints as *D*;
2. $C_n$ is contained in ${{\,\mathrm{conv}\,}}I_n$;
3. For any point *x* of $C_n$, the tangent of $C_n$ at *x* is orthogonal (in the Euclidean sense) to *x* if, and only if, *x* is the midpoint or an endpoint of $C_n$.

Consider the bump function

$$\begin{aligned} \varPsi (x) = {\left\{ \begin{array}{ll} \exp \left( -\frac{1}{1 - x^2}\right) &{}\quad \text {if}\;\; x\in (-1,1), \\ 0 &{}\quad \text{ otherwise. } \end{array}\right. } \end{aligned}$$

It is well known that $\varPsi$ is non-negative, smooth, its support is $[-1,1]$, and the only points in its support where the derivative is zero are $-1,1$ and 1/2.

Let the endpoints of $I_n$ be $(\cos \alpha _n,\sin \alpha _n)$ and $(\cos \beta _n,\sin \beta _n)$, where $\alpha _n<\beta _n$. Let $C_n$ be the curve

$$\begin{aligned} \varphi \mapsto \bigg (1-\varepsilon \varPsi \left( \frac{2}{\beta _n-\alpha _n}\left[ \varphi -\frac{\alpha _n+\beta _n}{2}\right] \right) \bigg )(\cos \varphi ,\sin \varphi ),\quad \varphi \in [\alpha _n,\beta _n], \end{aligned}$$

for some small $\varepsilon >0$.

Clearly, $C_n$ is a smooth curve, and if $\varepsilon$ is sufficiently small, then it is also strictly convex. Moreover, $C_n$ satisfies Property 1, as $\varPsi ^{\prime }(-1)=\varPsi ^{\prime }(1)=0$. If $\varepsilon$ is sufficiently small, then $C_n$ satisfies Property 2 as well. Finally, to verify Property 3, observe that the tangent of $C_n$ is orthogonal to $(\cos \varphi ,\sin \varphi )\in C_n$ if, and only if, the derivative of

$$\begin{aligned} \varphi \mapsto 1-\varepsilon \varPsi \left( \frac{2}{\beta _n-\alpha _n}\left[ \varphi -\frac{\alpha _n+\beta _n}{2}\right] \right) \end{aligned}$$

vanishes at $\varphi$. However, this is only the case at the midpoint and two endpoints of $C_n$.

The closed curve

$$\begin{aligned} L:=\left( {{\,\mathrm{bd}\,}}D{\setminus } (C\cup -C)\right) \cup \left( C_0\cup -C_0\right) \cup \bigcup _{n=1}^{\infty }\left( C_n\cup -C_n \right) \end{aligned}$$

is the boundary of a smooth, strictly convex, origin-symmetric planar body *K*, say. In order to identify the Auerbach points of *K*, first observe that if $x,y\in L$ form an Auerbach pair in *K*, then *x* and *y* are orthogonal in the Euclidean sense. (The converse does not hold, of course.) By this observation and Property 3, for each $n\in \mathbb {Z}^+$, the only Auerbach point in the relative interior of the arc $C_n$ is the midpoint of $C_n$. The same holds for $-C_n$. Again by the observation, all points of $C_0\cup -C_0$ are Auerbach points. Finally, again by the observation, the set of Auerbach points of $\left( {{\,\mathrm{bd}\,}}D{\setminus } (C\cup -C)\right)$ is the rotation of the previously described set of Auerbach points in $\left( C_0\cup -C_0\right) \cup \bigcup _{n=1}^{\infty }\left( C_n\cup -C_n \right)$ by an angle of $\pi /2$. It follows that *A*(*K*) is the union of two disjoint copies of the Cantor set and a countable set of isolated points.$\square$

## Proof of Proposition 2

We may assume that $0,1\in H$. Enumerate the components of $\mathbb {R}{\setminus } H$ as $I_0,I_1,\ldots$, where $I_0:=(-\infty ,0)$ and $I_1:=(1,\infty )$. We will recursively assign a real number $y_n$ to each open interval $I_n$. Let $y_0:=0$ and $y_1:=1$.

If $y_k$ has already been defined for all $k<n$, let

$$\begin{aligned} y_n:=\frac{1}{2}\left( \max _{\begin{array}{c} \ell<n\\ I_{\ell }<I_n \end{array}} y_{\ell } +\min _{\begin{array}{c} \ell <n\\ I_{\ell }>I_n \end{array}} y_{\ell } \right) , \end{aligned}$$

that is, we consider the two intervals with indices less than *n* just below and just above $I_n$, and $y_n$ is the average of the two values assigned to these two intervals.

We define a function *f* on $\mathbb {R}$ as follows. First, on $\mathbb {R}{\setminus } H$, let $f|_{I_n}=y_n$. To extend *f* to $\mathbb {R}$, we set

3 $$\begin{aligned} a_x:=\sup (-\infty ,x){\setminus } H,\quad \text {and}\quad b_x:=\inf (x,\infty ){\setminus } H. \end{aligned}$$

If $x\in H$ and $a_x=b_x$, then the left limit, $f(a_x-)$, of *f* at $a_x$ clearly equals the right limit $f(b_x+)$. Thus, the function

$$\begin{aligned} f(x):= {\left\{ \begin{array}{ll} y_n &{}\quad \text {if}\;\; x\in I_n;\\ f(a_x-)=f(b_x+) &{}\quad \text {if}\;\; x\in H \text { and } x=a_x=b_x;\\ f(a_x-)\dfrac{b_x-x}{b_x-a_x}+ f(b_x+)\dfrac{x-a_x}{b_x-a_x} &{}\quad \text {if}\;\; x\in H \text { and } a_x<b_x \end{array}\right. } \end{aligned}$$

is continuous, strictly increasing on *H*, and locally constant on $\mathbb {R}{\setminus } H$.

Finally, let $\mu _0$ denote the Lebesgue-Stieltjes measure corresponding to *f*, and $\mu _1$ the measure $\mu _1(A)=\lambda (A\cap H)$, where $\lambda$ is Lebesgue measure. Then $\mu =\mu _0+\mu _1$ is a continuous measure, and ${{\,\mathrm{supp}\,}}\mu \subseteq H$.

To show the reverse inclusion, let *I* be an open interval and assume that $I\cap H\ne \emptyset$. If $I\cap H$ is of positive Lebesgue measure, then $\mu (I)>\mu _1(I)>0$. Otherwise, *I* is intersected by at least two $I_k$. Indeed, if only one $I_k$ intersected *I*, then $I\cap H$ would be the union of at most two intervals, contradicting that *H* is perfect and of Lebesgue measure zero.

Since the values of *f* on distinct intervals $I_k$ are distinct, *f* is not constant on *I*, and hence, $\mu (I)>\mu _0(I)>0$, completing the proof of Proposition 2.

The total measure is $\mu (\mathbb {R})=\mu _0(\mathbb {R})+\mu _1(\mathbb {R})=1+\lambda (H)\in [1,2]$, and thus $\nu =\mu /\mu (\mathbb {R})$ is a probability measure with the desired properties. $\square$
